# Human-Based Advanced *in vitro* Approaches to Investigate Lung Fibrosis and Pulmonary Effects of COVID-19

**DOI:** 10.3389/fmed.2021.644678

**Published:** 2021-05-07

**Authors:** Mirjam Kiener, Nuria Roldan, Carlos Machahua, Arunima Sengupta, Thomas Geiser, Olivier Thierry Guenat, Manuela Funke-Chambour, Nina Hobi, Marianna Kruithof-de Julio

**Affiliations:** ^1^Department of Pulmonary Medicine, Inselspital, Bern University Hospital, University of Bern, Bern, Switzerland; ^2^Department for BioMedical Research DBMR, Urology Research Laboratory, University of Bern, Bern, Switzerland; ^3^Alveolix AG, Swiss Organs-on-Chip Innovation, Bern, Switzerland; ^4^Department for BioMedical Research DBMR, Department of Pulmonary Medicine, Inselspital, Bern University Hospital, University of Bern, Bern, Switzerland; ^5^Organs-on-Chip Technologies, ARTORG Center for Biomedical Engineering, University of Bern, Bern, Switzerland; ^6^Department of General Thoracic Surgery, Inselspital, Bern University Hospital, University of Bern, Bern, Switzerland; ^7^Organoid Core, Department for BioMedical Research, University of Bern, Bern, Switzerland

**Keywords:** COVID-19, interstitial pulmonary fibrosis, SARS-CoV-2, alveolar regeneration, organoids, lung-on-chip, precision-cut lung slices, *in vitro* lung models

## Abstract

The coronavirus disease 2019 (COVID-19) pandemic has caused considerable socio-economic burden, which fueled the development of treatment strategies and vaccines at an unprecedented speed. However, our knowledge on disease recovery is sparse and concerns about long-term pulmonary impairments are increasing. Causing a broad spectrum of symptoms, COVID-19 can manifest as acute respiratory distress syndrome (ARDS) in the most severely affected patients. Notably, pulmonary infection with Severe Acute Respiratory Syndrome coronavirus 2 (SARS-CoV-2), the causing agent of COVID-19, induces diffuse alveolar damage (DAD) followed by fibrotic remodeling and persistent reduced oxygenation in some patients. It is currently not known whether tissue scaring fully resolves or progresses to interstitial pulmonary fibrosis. The most aggressive form of pulmonary fibrosis is idiopathic pulmonary fibrosis (IPF). IPF is a fatal disease that progressively destroys alveolar architecture by uncontrolled fibroblast proliferation and the deposition of collagen and extracellular matrix (ECM) proteins. It is assumed that micro-injuries to the alveolar epithelium may be induced by inhalation of micro-particles, pathophysiological mechanical stress or viral infections, which can result in abnormal wound healing response. However, the exact underlying causes and molecular mechanisms of lung fibrosis are poorly understood due to the limited availability of clinically relevant models. Recently, the emergence of SARS-CoV-2 with the urgent need to investigate its pathogenesis and address drug options, has led to the broad application of *in vivo* and *in vitro* models to study lung diseases. In particular, advanced *in vitro* models including precision-cut lung slices (PCLS), lung organoids, 3D *in vitro* tissues and lung-on-chip (LOC) models have been successfully employed for drug screens. In order to gain a deeper understanding of SARS-CoV-2 infection and ultimately alveolar tissue regeneration, it will be crucial to optimize the available models for SARS-CoV-2 infection in multicellular systems that recapitulate tissue regeneration and fibrotic remodeling. Current evidence for SARS-CoV-2 mediated pulmonary fibrosis and a selection of classical and novel lung models will be discussed in this review.

## Introduction

Coronavirus disease 2019 (COVID-19) is a zoonotic disease caused by the novel Severe Acute Respiratory Syndrome coronavirus 2 (SARS-CoV-2). SARS-CoV-2 is the seventh coronavirus known to infect humans. Human coronavirus strains HKU1, OC43, NL63 and 229E cause mild symptoms similar to the common cold, while SARS-CoV and Middle East Respiratory Syndrome coronavirus (MERS-CoV) can result in severe viral pneumonia with a high mortality and have been responsible for two epidemic outbreaks in the twenty-first century (1). Compared to SARS-CoV and MERS-CoV, SARS-CoV-2 is more easily transmitted from human to human which has allowed it to evolve into a worldwide pandemic (2). SARS-CoV-2 enters the human body via the respiratory tract and reaches its initial main target organ, the lung. About one-fourth to one-third of hospitalized patients develop severe complications and require treatment in the intensive care unit for ~10 days or longer (3, 4), which risks a global collapse of the health care system. Countermeasures including curfews to limit the spread of SARS-CoV-2 have caused dramatic economic losses (5). Despite improved management of critically ill patients (6) this situation can only be resolved by effective treatment strategies and COVID-19 vaccines. Four COVID-19 vaccines have already been approved in Europe (ema.europa.eu) and various other vaccines are currently being developed or have entered late-phase clinical trials (7). In parallel, inhibitory compounds are tested for re-purposing (8, 9). *In vitro* models of the respiratory tract have significantly contributed to screening for promising drug candidates such as remdesivir, camostat, imatinib, and Retro-2.1 and have helped elucidating the molecular mechanisms of host-pathogen interactions in more detail (10,15). Increasing knowledge about the course of COVID-19 raised concerns regarding its long-term consequences. Experts warn that SARS-CoV-2 might cause long-lasting or persisting interstitial pulmonary fibrosis, an incurable clinical condition marked by abnormal fibrogenesis in the alveolar wall resulting in a progressive reduction of pulmonary function and gas exchange in the lung (16). Recent studies show that severe or critically ill COVID-19 survivors have reduced diffusion capacity and oxygenation levels compared to mildly or moderately sick patients 4 months after infection (17). Whether these impairments resolve, remain or evolve into persisting pulmonary fibrosis is currently unknown.

This review focuses on the clinical course of COVID-19 in the lung and relates the pathology to the underlying molecular biology. Furthermore, we will discuss interstitial pulmonary fibrosis, with idiopathic pulmonary fibrosis (IPF) as the worst example, and how COVID-19 may lead to pulmonary fibrosis. Finally, we will review available *in vivo* and *in vitro* models of lung fibrosis and SARS-CoV-2 infection to propose the most suited advanced *in vitro* models for studying COVID-19-associated pulmonary fibrosis.

## Pathogenesis of COVID-19 in the Lung

### Fundamental Processes of Breathing: The Biology and Regeneration of the Lung Epithelium

**Figure 1:**
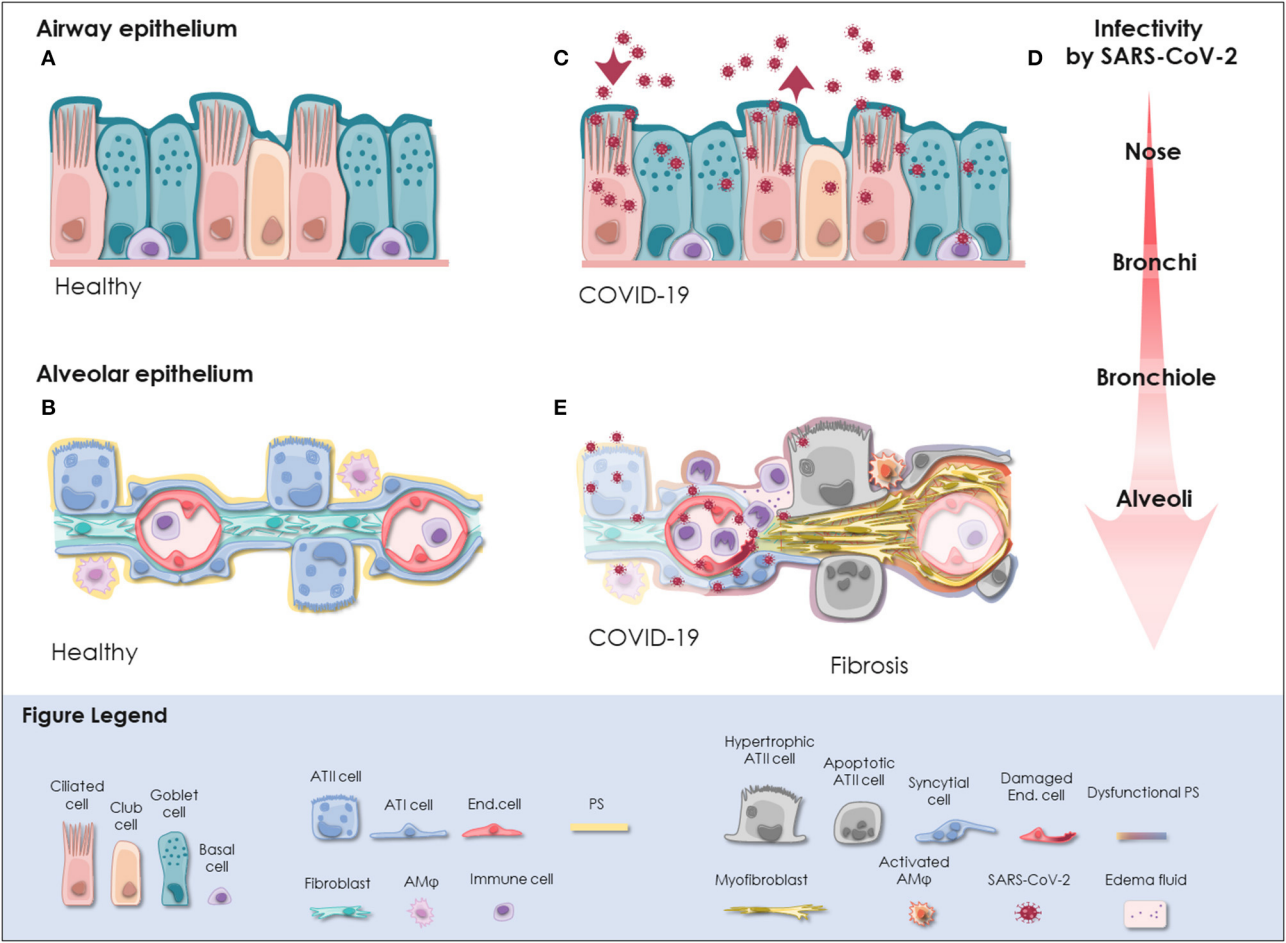
SARS-CoV-2 infection in the respiratory tract. **(A)** In the pseudostratified epithelium of the airways, secretory goblet, and club cells produce mucus, which is transported by ciliated cells to clear trapped particles and protect the lung from micro-injuries and infection. Basal cells reside at the lamina propria and comprise progenitor cells. The composition and frequency of the individual cell types is variable among the distinct anatomical sites in the nose, trachea, bronchi, and bronchioles. **(B)** The alveolar epithelium is specialized for gas exchange with flattened ATI cells forming an ultra-thin (~2 m) epithelial-endothelial barrier allowing oxygen and CO_2_ diffusion. Cuboidal ATII cells are considered as progenitor cells of ATI cells and fulfill vital functions by the production of pulmonary surfactant (PS), which lowers surface tension and prevents alveolar collapse. Lung fibroblasts are essential to maintain the ATII stem cell niche. Resident alveolar macrophages (AM) and immune cells defend the epithelium from infection. **(C)** SARS-CoV-2 initially infects the airway epithelium. The virus can efficiently replicate in ciliated and secretory cells resulting in the shedding of high viral titers and mild to moderate COVID-19 symptoms. **(D)** The respiratory epithelium exhibits differential susceptibility to SARS-CoV-2 infection. In correlation with ACE2 expression, SARS-CoV-2 infection is most efficient in the upper airways, particularly in the nasal epithelium. Infectivity gradually decreases toward the alveoli. However, when SARS-CoV-2 reaches the alveoli it can result in severe manifestation of COVID-19. **(E)** Upon reaching the alveoli, SARS-CoV-2 infects alveolar epithelial cells and endothelial cells and causes viral pneumonia. Cytopathic effects of SARS-CoV-2 are evident as syncytial and apoptotic alveolar epithelial cells resulting in the breakdown of pulmonary surfactant and barrier integrity. In some patients, alveolar damage culminates in life-threatening microvascular activation and an imbalanced immune response. Tissue regeneration takes place already during acute COVID-19 as indicated by fibrin deposition, ATII cell hyperplasia and alveolar wall thickening. Moreover, severely ill COVID-19 patients exhibit radiological signs of fibrosis even months after recovery indicative for the induction of COVID-19-associated fibrosis. ACE2, angiotensin-converting enzyme 2; ATI cell, type I alveolar epithelial cell; ATII cell, type II alveolar epithelial cell; COVID-19, coronavirus disease 2019; End. cell, endothelial cell; AM, alveolar macrophage; PS, pulmonary surfactant; SARS-CoV-2, severe acute respiratory syndrome coronavirus 2.

The respiratory tract is continuously exposed to inhaled particles and pathogens. Therefore, it is lined by a highly specialized epithelium, which can be divided into conducting airways and alveoli based on their location and primary function. The pseudostratified epithelium in the proximal airways harbors secretory club and goblet cells, which produce a protective layer of mucus toward the lumen. The terminally differentiated ciliated cells convey the mucus layer upwards to clear trapped particles. Basal cells are able to differentiate into secretory or ciliated cells and are therefore considered to represent the progenitor cells of the airway epithelium, though most cell types of the airway epithelium are highly plastic [(18); Figure 1A]. On the distal end, the conducting airways branch into bronchioles and ultimately in the alveoli. These sac-shaped units represent one of the largest body surfaces in constant contact with the environment essential for efficient gas exchange. About 95% of the alveolar surface is covered by highly specialized flattened type I alveolar epithelial (ATI) cells (19). They form an ultra-thin epithelial-blood barrier with the pulmonary microvasculature endothelial cells, supporting efficient oxygen and CO_2_ passive diffusion (20). Together with ATI cells, type II alveolar epithelial (ATII) cells are the main constituents of the highly differentiated alveolar epithelium, which closely interacts with surrounding cells in the niche including alveolar macrophages (AM), microvascular cells, and fibroblasts (Figure 1B).

ATII cells are cuboidal cells often located at the edges of the alveolar sacs and, as opposed to the flat and large ATI cells, account for a small fraction of the alveolar surface. ATII cells produce pulmonary surfactant, a lipid-protein complex with exceptional surface tension lowering properties (21). By doing so, they sustain the breathing function and protect the delicate alveolar structure from collapsing upon exhalation (21). ATII cells also have a role in innate immunity and take part in surfactant recycling. But most importantly, ATII cells are capable of self-renewal and differentiation into ATI cells, which allows re-epithelization upon injury [reviewed in (22)].

Already in 1977, Mason and Williams termed ATII cells as the defender of the alveolus for their central role in lung homeostasis (23). However, what exactly defines an ATII cell has been a matter of discussion for years (24). *In vitro*, isolated human ATII cells behave as facultative stem cells giving rise to alveolar organoids containing multiple cell types (25, 26). Recent studies have suggested that different ATII subtypes may coexist within what has been classically considered as ATII cells, including the proposed alveolar epithelial progenitors comprising TM4SF1^+^ cells which are highly responsive to Wnt signaling (26).

Rising evidence supports the role of other cell types in alveolar tissue repair together with ATII cells. A subset of Hopx^+^ ATI cells has been suggested as a source of ATII cells via transdifferentiation upon injury (27). Other studies have proven that a rare basal-like p63^+^ Krt5^+^ epithelial cell population migrates to sites of injury in the distal lung to re-create the damaged barrier in the mouse [reviewed in (28)]. In humans, such a population has not been found to date, but bronchiolization is a common histologic finding after injury. In addition, the contribution of basaloid cells in the repair process is supported by the finding of basaloid cells in the damaged areas of patients suffering from IPF, an aggressive form of progressive interstitial pulmonary fibrosis with unknown cause, although several risk factors have been identified [reviewed in (28)]. Recently, EpCAM^+^ CD73^+^ epithelial cells, which localize at the basal membrane of the respiratory and alveolar epithelium, have also been suggested as progenitors for both, pseudostratified mucociliary and mature alveolar epithelium in the postnatal and adult human lung (29).

Further, the contribution of stromal cells to ATII cell stemness maintenance and tissue repair cannot be neglected. Lung fibroblasts have been shown to support progenitor ATII cell characteristics *in vitro* and *in vivo* in mice (25, 30, 31) and human (25), underscoring the relevance of Wnt signals as determinants for ATII cell fate. On the other hand, fibroblasts, and myofibroblasts are also responsible for extracellular matrix (ECM) deposition and wound closure upon alveolar injury (32).

In summary, repair in the alveolar epithelium is characterized by an acute inflammatory phase, progenitor differentiation and migration, wound closure and finally, resolution (33). Upon injury, ATII cells behave as facultative stem cells and activate their regenerative response becoming hyperplastic. These ATII cells will either self-renew, migrate to the site of injury and differentiate into ATI cells, or undergo apoptosis. These processes depend on the balance of different mediators and a complex cell-cell crosstalk in which stromal cells and AM are crucial players (34). Some studies point at the pro-inflammatory and oxidative environment as a driving force for differentiation and repair in the mouse [reviewed in (22)], with Wnt signaling as a key regulator for ATII cell differentiation (35). Further, the relevance of ATII cells in the repair process is highlighted by studies in which ATII-targeted damage or cellular intrinsic alterations, rather genetic or due to aging, lead to aberrant tissue remodeling (36, 37).

It is also important to consider that the lung is subject to mechanical stress and deformation which is essential for several key biological events such as lung development (38) and pulmonary surfactant secretion (39,41). The correlation between alveolar inflation to the corresponding increase in alveolar surface area is still debatable. Nevertheless, during restful breathing, also termed as tidal breathing (defined as 4080% of TLC, total lung capacity), alveolar linear strain has been suggested to go from 4 to 10% (42,45), up to even higher than 20% during exercise or deep sighs (42, 43, 45, 46). Hence, local mechanical tension and stiffness changes which occur along the repair process converge with the forces supporting breathing (38). In fact, breathing-like cyclic strain has been proven to influence the regenerative epithelial response as shown by wound closure experiments *in vitro* (47,51). Mechanical ventilation with high tidal volumes, on the other hand, has been observed to amplify lung damage in animal models and in ventilated patients suffering from different respiratory pathologies (52, 53). In fact, mechanical stress has been suggested as an important factor for fibrogenesis (54). Considering this, protective ventilation protocols have been adopted to prevent ventilation-associated lung injury in COVID-19 patients (55).

Besides stretch, the alveolar niche sustains other mechanical forces such as shear stress and surface tension. At the alveolar epithelium, surface tension, and the so-called interfacial stress dominate particularly at low volumes (45). These forces stem from the continuous change in area exposed to the air, its associated fluid oscillation and cell-induced deformation (45). Interfacial stress alone has been observed to be deleterious for ATII cells *in vitro*, however, it has been also proven to constitute a powerful signal for pulmonary surfactant release in addition to cyclic stretch (56, 57). Pulmonary surfactant efficiency in lowering surface tension is tightly associated to its lipid and protein composition, which adapts very quickly to meet different respiratory demands (58, 59), and has been suggested to be refined along breathing cycles in a mechanism assisted by surfactant proteins (60,64). In the context of surfactant exhaustion, higher surface tension may then act as a trigger for further surfactant release to restore alveolar homeostasis. This system fails in pathological conditions in which aberrant surfactant composition (65,67) contributes to associated higher surface tension and repetitive tissue damage (54), thus stressing the relevance of surfactant and ATII as a secreting cell in addition to its role in repair.

Altogether, this evidence highlights the complexity of alveolar epithelial repair and the central role played by ATII cells. Hence, we speculate that targeted ATII cell injury such as that caused by SARS-CoV-2 infection may increase alveolar susceptibility to injury and aberrant tissue repair, with severe long-term consequences even after disease resolution.

### Molecular Mechanisms of SARS-CoV-2 Infection in the Lung

The initial step of coronavirus infection involves binding of the viral spike (S) protein to the compatible receptor on the surface of the target cell (68). Like the closely related SARS-CoV, SARS-CoV-2 uses angiotensin-converting enzyme 2 (ACE2) as an essential entry receptor into human cells. In contrast to SARS-CoV, the receptor binding domain (RBD) of SARS-CoV-2 S protein even exhibits higher binding affinity for human ACE2 (69,72) but seems to be less exposed possibly enabling immune evasion (70). As a consequence, ACE2 affinity of SARS-CoV and SARS-CoV-2 full-length S protein is comparable enabling both viruses to attach to human ACE2 but not to other coronavirus entry receptors such as aminopeptidase N (APN) and dipeptidyl peptidase 4 (DPP4) (11, 73, 74).

Subsequently, proteolytic cleavage of the S protein exposes the fusion domain and enables virus entry into the host cell (75). Multiple proteases can fulfill this function such as transmembrane protease serine subtype 2 (TMPRSS2) and cathepsin B/L in case of SARS-CoV (76,79) or TMPRSS2, cathepsin L, and Furin for MERS-CoV (80, 81). Strong evidence for SARS-CoV-2 S protein priming by TMPRSS2 and cathepsin L has been gathered *in vitro* (11, 69, 82).

Therefore, cells co-expressing ACE2 and TMPRSS2 can potentially be infected by SARS-CoV-2. Single cell RNA sequencing data analysis has revealed ACE2 expressing cells in multiple organs, though it is generally expressed at low levels. This suggests that ACE2 expression is the limiting factor for SARS-CoV-2 infection (83, 84). However, enriched expression of ACE2 protein and co-expression with TMPRSS2 potentially renders alveolar epithelial cells and enterocytes particularly vulnerable to SARS-CoV-2 (83,85). Accordingly, SARS-CoV-2 RNA is detected prominently in the respiratory tract but occasionally also in the feces and blood of COVID-19 patients (86,88). In the respiratory tract, SARS-CoV-2 is detected in diagnostic samples and tissue specimens from different anatomical sites implying that it can replicate throughout the airway and lung epithelium (74, 89,91). Despite overall low ACE2 expression levels in the respiratory tract, about 20% of lung cells have been found to express ACE2 mRNA (82). The highest levels of ACE2 are reached in the nasal epithelium and gradually decrease from the proximal airways toward the distal lung (82, 83, 92). Accordingly, viral yields are higher in nasal swabs than throat swabs indicating that the nasal epithelium is the initial site of SARS-CoV-2 infection, replication, and shedding (74). The infection can propagate further as ACE2 and TMPRSS2 expression is found throughout the airway epithelium, particularly in ciliated and secretory cells (83, 92). Correspondingly, ciliated cells and goblet cells in the trachea and bronchi are efficiently infected by SARS-CoV-2, whereas basal cells are permissive for SARS-CoV-2 to a lower extent [(13, 14, 82, 93, 94); Figure 1C]. The finding that SARS-CoV-2 does not infect ciliated cells of distal lung organoids but exhibits a strong tropism for club cells seems contradictory (95). However, the cell tropism of SARS-CoV-2 might shift among different anatomical sites given the highly variable infection efficiencies reported for *in vitro* cultured ciliated, goblet and club cells (14, 82, 95). Moreover, ACE2 is upregulated upon interferon (INF) stimulation to protect the tissue during acute lung injury (96). Despite inducing an imbalanced immune response and delayed IFN signaling (97), we cannot rule out that SARS-CoV-2 infection itself might trigger INF-mediated upregulation of ACE2 promoting infection. Taken together, it is likely that SARS-CoV-2 initially infects and replicates in the nasal epithelium, particularly in ciliated cells, achieves high titers in the proximal airways and reaches the alveoli by aspiration through the airways [(74, 82, 90); Figure 1D].

In the alveoli, SARS-CoV-2 can be detected in ATI and ATII cells, endothelial cells and immune cells of deceased COVID-19 patients, which is in line with experimental findings from 3D *in vitro* models (82, 95, 98,101). Infection of ATI cells, endothelial cells and alveolar immune cells presumably results in a disturbed immune response and persistent inflammation (98,100). However, based on the analysis of single cell RNA sequencing datasets and *in vitro* infectivity experiments it has been suggested that ATII cells represent the primary target of SARS-CoV-2 in the alveoli [(82, 83, 92, 95, 100,102); Figure 1E]. Notably, increased susceptibility of an ATII cell subpopulation has been consistently reported by *in vitro* studies (99, 101). Gene expression profiling revealed an apoptotic signature and a strong downregulation of ATII-specific genes including surfactant proteins in heavily infected ATII cell models (101,103). In line with *in vitro* data, the induction of apoptotic pathways paralleled by a significant downregulation of surfactant protein transcripts is also apparent in ATII cells of COVID-19 patients (103) suggesting that SARS-CoV-2 infection results in the loss of ATII cell identity and function. Ultimately, this potentially leads to reduced surfactant production and consequently, alveolar collapse, massive tissue damage, and scaring (54). Therefore, further investigations on this fatal course of the disease are critical. To date, it is unclear whether these highly infected cells secrete viral particles, what are the immunological and clinical consequences and why a subpopulation of ATII cells seems to be more vulnerable than others. Possibly, SARS-CoV-2 relies on different entry mechanisms among different cell types and subsets. For example, it has been shown that TMPRSS2 is critical for SARS-CoV-2 entry in ATII cells but cathepsin B/L seems to be dispensable (102). Furthermore, as opposed to SARS-CoV, SARS-CoV-2 can exploit a wider range of host factors for cell entry, which can act synergistically with initial ACE2 attachment and TMPRSS2 cleavage. Detailed resolution of the sequence and structure of SARS-CoV-2 S protein has revealed only 73% similarity to SARS-CoV S protein RBD (104) and the presence of a multibasic site at the S1/S2 subunit boundary of SARS-CoV-2 S protein, which creates a novel furin cleavage site (70, 71, 105). Accordingly, furin overexpression enhances SARS-CoV-2 uptake (82) and has a cumulative effect with TMPRSS2 and cathepsin L on virus entry (70). Processing of the SARS-CoV-2 S protein by furin or other members of the proprotein convertase subtilisin kexin (PCSK) family might be highly relevant during SARS-CoV-2 infection of ATII cells as a recent meta-analysis of human lung single-cell RNA sequencing datasets has demonstrated significant co-expression of ACE2 and PCSK proteases in lung cells (85). Importantly, S protein processing by furin generates a RRAR motif at the S1 C-terminus which is able to bind to Nuropilin-1 (NRP1) and Nuropilin-2 (NRP2) (106). While ACE2 is still required for initial attachment of the virus to the cell surface, NRP1 depletion significantly reduces SARS-CoV-2 uptake (106). Notably, deletion of the multibasic S1/S2 site in SARS-CoV-2 S protein decreases the infection efficiency in human lung cells (105) and attenuates pathogenicity in animal models (107). Whether this is due to the loss of interaction with NRP1 and NRP2 remains to be demonstrated. However, NRP1 and NRP2 are upregulated in the lung tissue of COVID-19 patients (108), which might promote disease progression.

These data indicate that ACE2 expression is critical for SARS-CoV-2 infection and mediates initial attachment (71, 105). At the same time, activation of SARS-CoV-2 S protein by TMPRSS2, cathepsin L, and furin allows it to interact with surface molecules other than ACE2 (70, 106). This is likely to confer wider tissue tropism and promotes transmissibility of SARS-CoV-2. Once SARS-CoV-2 has entered the host cell and released its positive-sense single-stranded RNA genome into the cytoplasm, viral non-structural proteins are translated to generate the viral replication and transcription complex (RTC). Furthermore, coronavirus proteins hijack the translation machinery of the host cell and favor translation of viral mRNA over cellular mRNA, inhibit the innate antiviral IFN response and interfere with normal cell function (109). Infection of alveolar cells potentially results in the most critical disease manifestation due to abrogation of ATII cell function and stimulation of an inflammatory response.

### Acute Pathologic Manifestation of COVID-19 in the Lungs

SARS-CoV-2 infection results in a complex symptomatology associated with mild, moderate and severe illness or might even take an asymptomatic course (110,113). In non-hospitalized patients testing positive for SARS-CoV-2 infection, the most prevalent symptoms include cough, dyspnea, loss of smell or taste, fever and chills, myalgia, headache, body aches, sinus congestion, sore throat, nausea, diarrhea and dizziness (110, 111). Surprisingly, subclinical lung opacities and diffuse consolidation have been detected on computed tomography (CT) scans in more than half of asymptomatic COVID-19 patients (112, 114). Moreover, histologic alterations in the alveoli including edema, protein and fibrin exudate, ATII cell hyperplasia and fibroblast proliferation, inflammatory clusters and multinucleated giant cells have been reported in two pre-symptomatic cases of COVID-19 (115). Radiologic lung abnormalities seem to resolve in mildly to moderately symptomatic COVID-19 patients but the regeneration process in these patients is scarcely studied (116, 117).

In contrast, about one-third of patientsmainly elderly men with underlying comorbiditieshave a severe course of the disease with a high case fatality rate (3, 4, 118,121). Host factors rather than viral factors seem to be the significant determinants for disease severity. Pre-existing comorbidities, old age, male sex, and blood group other than O have been associated with a higher susceptibility to SARS-CoV-2 and risk for a severe disease course (118, 119, 122, 123). Furthermore, clinical parameters at hospitalization are critical predictors of severe illness. These include elevated levels of coagulation markers (e.g., D-dimers) in the blood (124) and lymphocytopenia, which correlates with increased interleukin (IL)-6 and IL-8 levels and a higher risk of cytokine storm (121). Autopsies have revealed that SARS-CoV-2 infects multiple organs including upper airways, lung, heart, kidney, the vasculature and the brain (125) and as a consequence can manifest extra-pulmonary [reviewed in (126)].

However, most commonly severe COVID-19 patients develop viral pneumonia and suffer from fever, fatigue, dry cough, myalgia and dyspnea (3, 4). In these patients, SARS-CoV-2 replicates in the upper airways and the distal lung, where it causes life-threatening damage to the alveoli (90, 98, 125, 127, 128). Nearly all hospitalized COVID-19 patients present with ground-glass opacities with or without consolidation on chest CT scans that gradually worsen before death (3, 4, 119, 120, 129). Critically ill patients usually develop acute respiratory distress syndrome (ARDS) (3, 4). ARDS can be provoked by various direct or indirect pulmonary insults. Infection, including viral infection, is a major cause for ARDS, being pneumonia the most common underlying pathology (130). ARDS is defined as the clinical manifestation of diffuse alveolar damage (DAD) (131). Correspondingly, typical histologic patterns of DAD including hyaline membrane formation, fibrin exudates, syncytial alveolar epithelial cells, diffuse ATII cell hyperplasia and the replacement of ATI cells by cuboidal ATII-like cells are apparent in the lungs of deceased COVID-19 patients [(100, 120, 125, 129); Figure 1E]. The recent description of two differential pathologic patterns in the lungs of deceased COVID-19 patients suggests that both, direct cytopathic effect of SARS-CoV-2 and a deleterious inflammatory immune response, can cause fatal alveolar damage (132). As a consequence, marked hypoxia develops and results in the enlargement of the pulmonary vasculature, blood vessel activation and coagulopathies with formation of micro-thrombi in multiple organs (6, 98, 108, 125). About 2% of hospitalized COVID-19 patients ultimately succumb to the disease with respiratory or multi-organ failure as a major cause of death (127, 128, 133). However, it is currently not known whether severely affected COVID-19 survivors will fully recover or may suffer from complications in the resolution phase of ARDS. First results after 4 months indicate that the diffusion capacity is reduced in COVID-19 patients after severe or critical disease (17).

## Pulmonary Fibrosis: A Long-Term Complication of COVID-19?

### Alveolar Damage as a Cause of Interstitial Pulmonary Fibrosis

It has been reported that ARDS can lead to lasting physical impairment after 5 years of follow up (134), including fibrotic pulmonary changes as a consequence of abnormal wound healing (135). Acute alveolar damage (e.g., from viral infection) is followed by the activation of inflammatory and apoptotic responses (136, 137). The alveolar epithelial cell damage triggers a cascade of reactions, including the release of pro-inflammatory cytokines, to activate local immune responses and controlled fibroblast proliferation as well as interstitial fibrogenesis, to initiate primary wound healing mechanisms (138, 139). These effects will normally be reconstituted by recovery of the basal lamina, re-epithelialization of the alveolar epithelium (140), and the degradation as well as clearance of ECM proteins (141). A precise and controlled repair mechanism following alveolar damage is crucial to terminate progression of the lung remodeling toward pulmonary fibrosis.

However, sustained alveolar injuries together with possible intrinsic factors, such as genetic mutations [e.g., MUC5B, SFTPC, TERT/TERC or TERF-1; (142,145)] or an accelerated aging phenotype (146), can impair the capability of alveolar epithelial cells to proliferate and orderly cover the defect. This provokes chronic alveolar damage that can eventually trigger an uncontrolled fibrotic response (147). This impaired wound healing can generate a disequilibrium in favor of the pro-fibrotic factors such as tumor necrosis factor alpha (TNF-), platelet-derived growth factor (PDGF) or transforming growth factor beta (TGF-), which will mediate the development and further progression of lung fibrosis (148). Particularly, TGF- has an essential role in activating fibrotic mechanisms, inducing the perpetuation of exaggerated wound repair (149).

The aberrant wound healing response can lead to additional loss of alveolar epithelial cells by apoptosis (150), induce lung fibrosis by activation of a pro-fibrotic profile in macrophages (151) and maintain the unruly activation and regulation of fibrotic lung fibroblasts mediated by TGF- (152). This dysfunctional alveolar re-epithelialization favors the uncontrolled proliferation of lung fibroblasts and secretion of ECM proteins that consolidate the fibrotic change (153). Indeed, viral lung infections can trigger DAD on top of interstitial lung diseases (ILD) which is a common histological feature in some stages of ILD progression (154, 155). Given the development of DAD that manifests as ARDS in severely sick COVID-19 patients, it remains to be investigated whether the alveolar wound healing response will eventually result in pulmonary fibrosis and in its worst form IPF.

### Emerging Evidence of COVID-19-Associated Lung Fibrosis

Long-term follow-up data on recovered COVID-19 patients is currently emerging and insights gained from earlier coronavirus epidemics can allow to predict likely scenarios. The first coronavirus epidemic of the twenty-first century has been caused by SARS-CoV, the causative agent of severe acute respiratory syndrome (SARS). SARS is an illness that shows typical infection-related symptoms, including fever and pneumonitis, with a recovery time in most patients after 12 weeks following the infection. Up to one third of SARS patients can develop severe pulmonary complications, requiring oxygen therapy (156). The acute phase of SARS starts with acute lung damage and edema, bronchiolar sloughing of ciliated epithelial cells and the deposition of hyaline-rich alveolar membranes, which clinically manifests with impaired oxygen exchange. A progressive phase during the following 25 weeks is characterized by fibrin deposition and infiltration of inflammatory cells and fibroblasts. In the last stage, after 12 months, pulmonary fibrosis consolidates with collagen deposition and fibroblast proliferation in the interstitial spaces (157,159).

The extent of fibrosis can be a sign of SARS severity and illness duration, as demonstrated in post-mortem studies (160, 161). Radiological features of fibrosis after SARS have been observed at 3 and 6 months after infection in around 30% of the cases, findings that have been confirmed by another study in survivors (162). Ground glass opacities were found 1 month after diagnosis in 45% of SARS patients, underlining the possibility to find early signs of fibrosis in those patients (163). Moreover, a patient's age can also be a critical risk factor in the fibrotic manifestation and long-term damage as older SARS patients have an increased risk for lung fibrosis (164).

Another coronavirus infectionthe Middle East respiratory syndrome (MERS), shows a similar clinical outcome as SARS. However, radiological abnormalities are more common in MERS (90100%) than SARS (60100%), and MERS patients have a higher incidence of ARDS with a higher case fatality rate (~36%). For both diseases, risk factors like age and male sex are associated with poorer disease outcomes (165).

Early evidence implies that, similarly to SARS and MERS, fibrotic remodeling and scaring occurs in the lungs of severely ill COVID-19 patients. An alarmingly large number of COVID-19 patients reported persistent symptoms, mainly fatigue and dyspnea, even months after first diagnosis in multiple independent surveys (166,168). In line, radiological signs of fibrosis become apparent as early as 3 weeks after diagnosis (169) and persist over months (170, 171). After 3 months, impaired diffusion capacity and persisting radiological abnormalities are observed in many survivors, while others recover completely (172,176). Further studies are ongoing whether radiological and functional impairments are chronic and even progressing. Worrisomely, lung autopsies of deceased COVID-19 patients have revealed the aberrant localization of mucus to the alveolar parenchyma, pathologic signs of proliferative DAD and thickening of the alveolar wall, particularly after a long severe phase (82, 120, 125). These findings suggest that COVID-19 induces lung abnormalities including cases with pulmonary fibrosis. Notably, virus-induced cell fusion has been shown to induce cellular senescence (177). Giant cells are a pronounced feature in COVID-19 lungs which might be due to furin-mediated cleavage of the SARS-CoV-2 S protein at the plasma membrane of ACE2 expressing cells resulting in syncytial alveolar epithelial cells (98). Potentially, this results in the acquisition of a senescent alveolar epithelial cell phenotype that can provoke inflammation and fibrosis (178,180). Moreover, intussusceptive angiogenesis occurs to a greater extent in pulmonary COVID-19 as compared to influenza A pneumonia, suggesting activation of tissue regeneration that follows similar patterns as in pulmonary fibrosis (108).

The possibility to use early anti-fibrotic strategies is currently being investigated (16). The principal feature of anti-fibrotic treatment is preventing the worsening of the disease by slowing down the fibrotic progression in established lung fibrosis, and potentially influencing the cytokine storm by anti-inflammatory effects of these drugs (181). Currently, some clinical studies are investigating both available anti-fibrotic treatments in patients with COVID-19 (recruiting phase): pirfenidone (NCT04282902, NCT04607928) and nintedanib (NCT04541680, NCT04619680). These results will provide us with new insights into the relevance of the fibrotic changes in COVID-19 and the effectiveness of anti-fibrotic treatment to improve the management of those patients in the future. In parallel, novel treatment strategies might be discovered *in vitro*, particularly in light of the recent advances in the field of *ex vivo* tissue cultures, lung organoids, and bioengineered microfluidic devices to study lung fibrosis.

## How Can We Study COVID-19-Associated Pulmonary Fibrosis?

### *In vivo* and *in vitro* Models of Pulmonary Fibrosis

**Figure 2:**
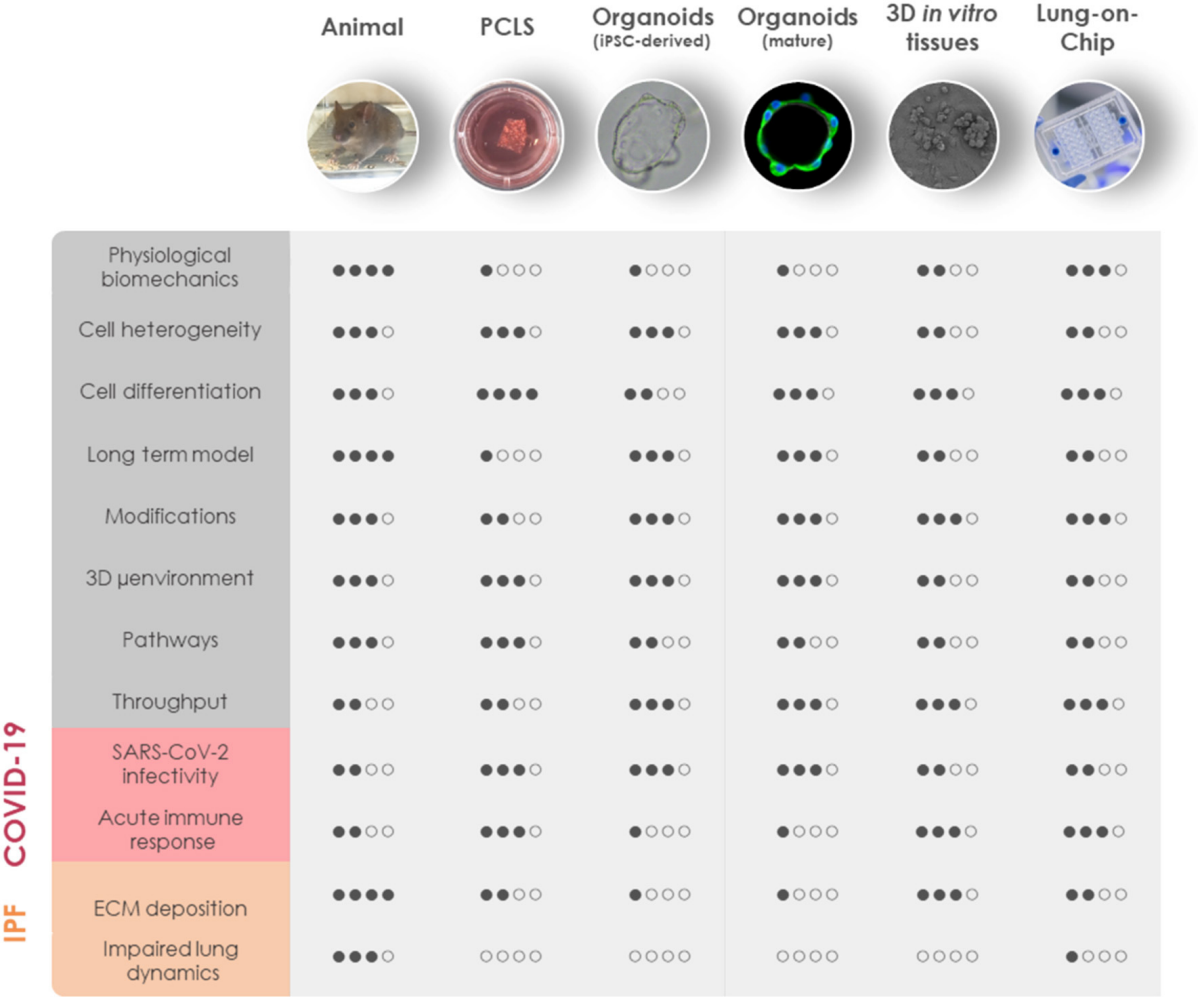
Comparison of *in vivo* and 3D *in vitro* lung models for COVID-19 and fibrosis research. General aspects of experimental animal models and advanced *in vitro* models including PCLS, iPSC-derived organoids, mature organoids, 3D *in vitro* tissues, and LOC are rated based on similarities to human physiology (physiological biomechanics, cell heterogeneity, cell differentiation, long-term model, and 3D microenvironment), genetic manipulation (modifications), the possibility for mechanistic investigations (pathways), and throughput capabilities (throughput). Their applicability to model the diseased state of the lung has been evaluated separately for COVID-19 and IPF. COVID-19, coronavirus disease 2019; IPF, idiopathic pulmonary fibrosis; iPSC, induced pluripotent stem cells; LOC, lung-on-chip; environment, microenvironment; PCLS, precision-cut lung slices; SARS-CoV-2, severe acute respiratory syndrome coronavirus 2.

Despite tremendous research efforts for pharmacological interventions over the past decade, pulmonary fibrosis remains one of the most challenging diseases to manage clinically. Although a single model is unable to mirror the progressive and irreversible nature of lung fibrosis, they provide valuable mechanistic insights into fibrogenesis. Animal experimental models have been widely used to understand the complex fibrotic responses and perform early pre-clinical testing for anti-fibrotic drugs. Among them, the bleomycin-induced pulmonary fibrosis model has been most widely used since the 1970's as the classical standard and best characterized *in vivo* fibrosis model (182). Contrary to human pulmonary fibrosis, bleomycin-induced fibrosis is temporary, and its inflammatory aspect justified criticism to accurately represent the pathophysiological process in IPF. Aside from bleomycin, fluorescein isothiocyanate (FITC) has also been widely used to induce experimental lung fibrosis which results in alveolar injury and acute fibrotic reaction that persist up to 24 weeks. Occupational exposure to environmental risk factors has been extensively associated with pulmonary fibrosis (183). Reports suggested that inhalation of silica and asbestos particles in rats results in fibrotic nodule formation which closely mimics prominent features of silicosis and asbestosis in humans with long-term occupational exposure (184, 185). Additionally, whole thorax irradiation in mice has been invaluable to study early inflammatory responses in radiation-induced lung fibrosis (186). It is well-established that IPF includes genetic predisposition affecting genes encoding e.g., surfactant protein-C (SP-C) (187), SP-A (188), Mucin-5B (MUC5B) (189), telomerase reverse transcriptase (TERT), and telomerase RNA component (TERC) (145). These known mutations have paved the way for genetically modified animal models of pulmonary fibrosis. Furthermore, intratracheal delivery of pro-fibrotic cytokines like TGF-1 (190), TNF- (191), and IL-1 (192) by adenovirus and lentivirus vectors have been extensively used to recreate mild early inflammation and rapid onset of lung fibrosis in mouse models. Despite the fact that animal models cannot fully recapitulate the complex, progressive and irreversible nature of lung fibrosis in humans, they remain the first line for preclinical testing in lack of appropriate alternatives. Nevertheless, animal models have been proven valuable for gaining a better mechanistic understanding of fibrogenesis, assessing lung function in the course of pulmonary fibrosis and performing pharmacokinetic studies (Figure 2).

However, most of our understanding of lung fibrosis stems solely from *in vitro* studies, typically relying on the activation of fibroblasts with pro-fibrotic cytokines in cellular models. Although *in vitro* fibrosis models represent a robust platform to study cell-specific responses to soluble cues in a controlled setting, cells *in vivo* are embedded in a complex 3D microenvironment with varied mechanical cues, cell-ECM interactions, differential polarity, and growth factor gradients. Given the strong involvement of fibroblasts and ECM in the pathology of fibrotic diseases, it is particularly important to maintain tissue architecture in human-derived models of fibrosis. Fibrotic tissue explants from patients suffering from a fibroproliferative skin disease, have been shown to retain viability for several days in *ex vivo* tissue culture, allowing to study molecular mechanisms of fibrosis and test novel therapeutic strategies (193). Recently, precision-cut lung slices (PCLS) have garnered increasing attention as a novel lung *ex vivo* fibrosis model. Overcoming the classical limitation for the study of human lung cells in 2D cell culture models, PCLS are able to spatially retain the native lung architecture along with fundamental ECM composition, stiffness and responsiveness together with viable lung resident cell populations [(194); Figure 2]. PCLS derived from healthy lung tissue resections closely mimic fibrotic-like changes including increased ECM deposition and alveolar remodeling when induced with a pro-fibrotic cocktail (195). A study in 2018 has reported that induction with TGF-1 resulted in increased deposition of collagen and ECM proteins in 2 mm^3^ sections of human lung parenchymal tissues within 1 week in culture (196). The close recapitulation of pathologic processes and the possibility to culture tissue from IPF patients allows to study drug responses *ex vivo*. Interestingly, nintedanib and pirfenidone exhibit distinct anti-fibrotic potential in mouse and human PCLS underscoring the need for human-derived models of IPF (197). Notch1 inhibition in PCLS derived from IPF patients has shown significant improvement in surfactant protein processing along with decreased ECM deposition and an overall reversal of fibrosis (198). In addition, a study for inhalation-based anti-fibrotic therapies has utilized advanced 3D printing technologies to develop a replica for Ear-Nose-Throat which has been connected to an *ex vivo* porcine respiratory tract within a sealed chamber. To mimic fibrosis-related alterations, mechanical properties of the lung parenchyma have been modified by reduction of lung compliance and passive ventilation which allowed them to analyze *in vivo* aerosol regional deposition in a fibrosis-mimicking environment (199). Although a key advantage in using human tissues is the exclusion of cross-species heterogeneity, the significant limiting factor of *ex vivo* tissue culture is the constant need for fresh tissues. Generally, they are not readily available as the tissues are mostly obtained from end-stage pulmonary fibrosis patients after lung transplantation or healthy surrounding tissue from tumor resections used for artificially induced early fibrotic changes *ex vivo*. Moreover, the complexities associated with long-term cultivation of the lung explants makes it difficult to standardize PCLS technique for high-throughput testing. Nevertheless, PCLS can be useful to investigate specific aspects of pulmonary fibrosis and viral infection directly in human lung tissue (Figure 2).

Efforts have been undertaken to generate easily accessible, controlled model systems that provide structural and cellular complexity but hold the possibility to increase the throughput. Different cell types in the lung contribute to the pathology of fibrosis and hence the choice of the cell system is an important consideration for *in vitro* studies (200). Moreover, recent studies have focused more on using mechanically tunable substrates over standard extremely stiff (10^6^ kPa) cell culture plastic dishes. Several studies have demonstrated that increased substrate stiffness directly influences (myo)fibroblast activation, differentiation and ECM deposition (201, 202). Instead, seeding fetal-derived fibroblasts on hydrogel beads to mimic the structure of alveolar sacs recreates the patchy areas of myofibroblast proliferation, contraction, and interstitial thickening upon TGF-1 stimulation as it is observed in IPF patients (203). Tests for novel IPF medication and mechanistic studies on fibroblast invasion of IPF patients have also been undertaken in self-assembled pneumospheres comprising heterogeneous cell populations (204). Additionally, biocompatible and biodegradable cross-linked polymer like Matrigel is a widely used substrate for 3D lung cell culture and organoid modeling for fibrosis. A recent study has analyzed transcriptional signatures of fibrotic lung organoids in order to identify aberrantly expressed genes (205). While multicellular organoids closely capture the minute details of cell-cell and cell-ECM interactions and physiological cellular organization they lack vasculature and air-liquid interface (ALI) [(206); Figure 2]. Recently, it has been shown that ALI promotes differentiation of human pluripotent stem cell (hPSC)-derived alveolar epithelial progenitor cells into ATII-like cells and reduces their transdifferentiation into ATI cells, which occurs in submerged cultures (207). Stimulation with a pro-fibrotic cocktail results in the loss of SPC^+^ ATII cells paralleled by an increase in MUC5B^+^ goblet-like cells mimicking the bronchialization process occurring in the alveoli of IPF patients (207). However, these models still lack biomechanical stimulation (Figure 2).

Advanced microfluidic technologies have been able to overcome these limitations with the development of lung-on-chip (LOC) devices (208). Organ-on-Chip technology is a new field emerging only recently as a system to model human tissues for the application in research and pharmacology. Despite the development of multiorgan systems, it remains challenging to apply the technique for gaining insights into systemic effects and standardize the models for pre-clinical testing. Moreover, microfluidic systems require the optimization of many factors such as the ECM, medium and scaffold material to support optimal cell growth. However, the complexity of Organ-on-Chip technology is also a chance allowing the modulation of a variety of biological, physical, and chemical factors in a controlled and closed system (209). A microfluidic device recreating the alveolar epithelium in ALI and in close contact with a microchannel, that is lined by endothelial cells and perfused with human full blood, has been employed to study pulmonary vascular inflammation and microthrombus formation (210). Furthermore, tiny wounds can be induced to the alveolar epithelium on chip either by trypsin or gastric-like content to mimic alveolar damage taking place in IPF (211) and wound-healing (212). Moreover, micro-tissues generated from human lung fibroblasts have been shown to exhibit enhanced contractility, stiffness and expression of alpha smooth muscle actin (-SMA), pro-collagen, and EDA fibronectin in response to TGF-, effects that have been reversed by treatment with pirfenidone (213).

Due to the importance of cyclic stretch for tissue regeneration after lung injury, Stucki et al. developed a breathing LOC model with primary human alveolar epithelial cells and lung endothelial cells. This system incorporates key mechanical forces of the alveoli including 3D cyclical stretch (corresponding to 8% linear strain) and surface tension (through the exposure to ALI) to recreate the complex alveolar microenvironment of the air-blood barrier [(214, 215); Figure 2]. Further advances in these models aiming at integrating pathophysiological stretch and introducing the often-neglected pulmonary surfactant warrant a bright future for accurate *in vitro* models of the alveolus. However, the availability of optimal biological material (e.g., high-quality tissue specimens from the relevant anatomical site, high cell viability, and physiological ECM composition) is often challenging and therefore requires further methodological advances in cell culture and tissue processing.

In summary, recent advancements in bio-engineered tissue and cell culture highlights promising platforms for lung fibrosis modeling and drug testing in a clinically-relevant setup (Figure 2). Importantly, lung fibrosis models that are compatible with SARS-CoV-2 infection models will enable investigations on the regenerative phase of COVID-19.

### Modeling SARS-CoV-2 Infection and Pathogenesis in the Respiratory Tract

*In vivo* models of viral infection integrate the full complexity of virulence factors, local and systemic immune responses and recovery. Therefore, animal models are particularly useful to test anti-inflammatory compounds and vaccines to combat infection (216). However, mice, the most widely available laboratory animals, are naturally resistant to SARS-CoV-2 infection (217, 218). The inability of SARS-CoV-2 to bind to murine ACE2 (74) poses the need to study COVID-19 in humanized mouse models expressing human ACE2 (217,220). SARS-CoV-2 infection in these mouse models results in weight loss, pneumonia and pathologic alterations in the lung tissue. However, the organ tropism and severity of symptoms varies among the models depending on the promoter to control human ACE2 expression. Mouse models expressing human ACE2 under the control of murine ACE2 develop rather mild symptoms and all animals spontaneously recover (217, 218). In contrast, severe pneumonia develops in mice expressing human ACE2 under the control of HFH4 or KRT18 promoter (219, 220). However, it remains arguable if these models correctly recapitulate SARS-CoV-2 tissue tropism given non-endogenous ACE2 expression patterns. Alternatively, mutation of the SARS-CoV-2 S protein or serial passaging in mice generates adapted virus to bind to murine ACE2 and infect the murine host (221, 222). These models might better resemble natural host-pathogen interactions in immunocompetent mice and result in mild pneumonia, however, it is unclear whether the mechanisms of mouse-adapted SARS-CoV-2 pathology can be translated to human.

Other animal species are naturally susceptible to SARS-CoV-2 (223). SARS-CoV-2 infects and replicates in ferrets but it is restricted to the upper respiratory tract allowing transmission studies but causing only mild symptoms (224, 225). Natural SARS-CoV-2 infection in golden hamsters and non-human primates involves the distal lung, however, results only in mild to modest pneumonitis and all infected animals spontaneously recover (226,231). Altogether, animal models recapitulate aspects of human COVID-19 such as an age-related risk to develop more severe disease as it has been demonstrated in mice and non-human primates (221, 222, 228, 230, 231). However, there are important differences between laboratory animal models and human COVID-19 pathogenesis (Figure 2). None of the available *in vivo* models captures the drastic hypoxia and associated coagulopathy, vascular inflammation and multi-organ failure as seen in severely ill COVID-19 patients. Mostly, SARS-CoV-2 infection takes a milder course in experimental animals or results in death by different pathologic mechanisms than in humans. This is likely due to a distinct distribution and affinity of ACE2 and TMPRSS2 for the SARS-CoV-2 S protein and fundamental differences in the immune system (232). Therefore, it is mandatory to complement *in vivo* data with findings garnered *in vitro* from human-derived models.

Essential knowledge about SARS-CoV-2 entry receptors, replication kinetics and cell-intrinsic immune response has been gained from *in vitro* studies using cell lines such as ACE2 overexpressing HeLa cells, the intrinsically IFN-deficient Vero E6 green monkey kidney cells or cancer cell lines (11, 74, 233). However, the relevance of SARS-CoV-2-induced gene expression changes in lung cancer cell lines such as A549 and Calu-3 remains arguable since these cells have lost the expression of Nkx-2.1, which is a master transcription factor for lung epithelial differentiation (234). Moreover, the lack of native ACE2 expression in some widely studied cell lines (e.g., HeLa, A549) and the absence of an intrinsic innate IFN response in Vero E6 cells hinders the assessment of normal physiological anti-viral responses (74, 235, 236). For this reason, the pathologic consequences of SARS-CoV-2 infection should ideally be studied in clinically relevant human cell systems.

Organoids generated from intestine, liver, microvasculature, kidney and airways are susceptible to SARS-CoV-2 infection. These studies have provided more comprehensive information on the SARS-CoV-2 target cell types and innate immune responses that are elicited by the virus (93, 237,240). Moreover, they provided functional evidence for the broad tissue tropism of SARS-CoV-2 as concluded earlier from the *in silico* analysis of ACE2 expression patterns among different organs and cell types (83). Due to the extraordinary difficulty to model the alveoli *in vitro* most studies aiming to elucidate the mechanisms of SARS-CoV-2 infection in its primary replication site have focused on the nasal, tracheal or bronchial airway epithelium. Bronchial organoids have been employed to identify target cell types in the upper airways and develop drug screening protocols. However, they do not support efficient SARS-CoV-2 infection (15, 101). This seems to be in disagreement with the higher ACE2 expression and susceptibility to SARS-CoV-2 infection in the upper airway epithelium as compared to alveoli (82). A possible explanation for this discrepancy might be the enrichment of basal progenitor cells in bronchial organoid cultures, which are not the primary target of SARS-CoV-2. Therefore, ALI cultures are more suitable to study SARS-CoV-2 in the upper airways. Human bronchial epithelial cells differentiate into functional ciliated and secretory cells in ALI cultures to form a pseudostratified epithelium capable of mucus production and cilia movement (241). They are efficiently infected by SARS-CoV-2 and produce high viral titers enabling functional studies and drug screening [(14, 82, 242); Figure 2]. In addition, a LOC model of the human bronchial epithelium under constant flow in the blood vessel chamber has recently been developed to study influenza A virus and SARS-CoV-2 infection and has led to the identification of candidate antiviral compounds (243). Hence, drug testing in a LOC device might further refine the number of candidate drugs (Figure 2).

In contrast, the alveoli are more challenging to reconstruct. Freshly isolated ATII cells rapidly transdifferentiate into ATI-like cells and are gradually lost in 2D *in vitro* cultures (244). A more stable ATII cell phenotype can be achieved by deriving ATII cells from induced hPSCs (245). These ATII cells can be maintained as organoids for prolonged cell culture but their main limitation is the fetal gene expression signature (10, 246). Seeding hPSC-derived ATII cells in ALI monolayers increases the degree of maturation, facilitates infection from the apical side and maintains the ATII cells, most likely due to the addition of the CHIR99021 Wnt agonist to the medium (102). SARS-CoV-2 infection elicits a rapid NF-B, TNF, IL-6, and IL-2 signaling driven inflammatory response in infected ATII cells but induces only a modest and delayed IFN response (10, 102). This indicates that hPSC-derived ATII cell models capture the intrinsic antiviral response of ATII cells but not the full spectrum of COVID-19. Nevertheless, hPSC-derived ATII-like ALI cultures are a useful tool to study early events of SARS-CoV-2 infection and discover compounds with anti-viral activity in the alveolar setting (247). Co-culture models of hPSC-derived lung organoids and hPSC-derived macrophages indicate that in this setting macrophages are essential producers of IFN- and drive protective or damaging immune responses (248). A major limitation of hPSC-derived alveolar models for high-throughput drug testing is their time- and cost-intensive derivation that involves a multi-step differentiation protocol [(249); Figure 2]. As a consequence, hPSC lines are usually generated from a few donors and maintained for the derivation of ATII cells, which results in a rather homogeneous genetic background. Therefore, they neglect individual genetic predispositions such as polymorphisms in IFN pathway genes or in mucus production and regeneration which might have an impact on disease severity and the fibrotic response after acute phase (250,253). A more heterogeneous cellular composition has been achieved by differentiating fetal lung-derived SOX2^+^SOX9^+^ bud tip progenitor cell organoids in 2D ALI cultures. They comprise alveolar-like and bronchial-like cell types and are readily infected by SARS-CoV-2 (254). However, this model meets similar limitations as hPSC-derived alveolar models due to the limited access to donor material and the derivation of differentiated bronchioalveolar ALI cultures from few organoid lines.

The patient-to-patient variability can be captured by adult stem cell derived alveolar organoid models. Alveolar organoids have been generated from HTII-280^+^-enriched ATII cells (101, 103) or mixed alveolar epithelial cells (13, 100). They maintain an ATII cell subpopulation during prolonged culture. In the intact organoids, ACE2 entry receptor faces the lumen while the basolateral side is exposed to the external milieu. In order to infect the organoids with SARS-CoV-2, the apical side has to be exposed either by apical-out polarization in suspension (95) or mechanical and chemical dissociation for the infection as single cells (13, 101) or as 2D monolayers (100). In contrast to hPSC-derived ATII cell monolayers, adult stem cell derived alveolar organoids nearly entirely lose the ATII cell population upon culture in 2D monolayers (100). Interestingly, it has been shown that ATII cells from dissociated organoids preferentially transdifferentiate into ATI cells in short-term submerged culture resulting in an alveolar-like epithelium. In contrast, maintaining the same cells in long-term ALI culture results in the replacement of alveolar epithelial cells by ciliated and goblet cells to form a pseudostratified airway-like epithelium (100). Notably, the authors describe sustained SARS-CoV-2 infection in submerged alveolar-like monolayers while ALI airway-like monolayers show a slow initial infection followed by exponential viral replication starting on day 2 post-infection. Furthermore, an innate immune response signature that resembles the gene expression signature in the lungs of deceased COVID-19 patients is induced in alveolar-like epithelium cultures (100). The authors conclude that the proximal airway components of the model are important for SARS-CoV-2 infectivity, while transdifferentiating alveolar epithelial cells subsequently recapitulate the host response (100). Multiple studies have reported the induction of an IFN response and upregulation of inflammatory (NF-B) pathways upon SARS-CoV-2 infection in alveolar organoids (13, 100, 101, 103). This characteristic allows to study IFN treatment, which has been administered to COVID-19 patients (255), in a physiologically relevant site. Interestingly, IFN treatment on alveosphere cultures induces apoptotic markers, upregulates ACE2 and TMPRSS2 expression and reduces the production of surfactant protein in ATII cells suggesting a potential positive effect on SARS-CoV-2 propagation. Nevertheless, IFN pre-treatment of alveospheres at lower doses impairs SARS-CoV-2 infection implying a preventive effect of IFN treatment in COVID-19 patients (103). Despite their utility for screening anti-viral and anti-inflammatory compounds, a major limitation of organoids is the lack of immune cells, a vascular-epithelial compartment and the ability to monitor epithelial barrier integrity (Figure 2).

LOC models have been applied in order to capture the complex physical and cellular microenvironment of the alveoli. They allow co-culture systems, temporal monitoring of trans-epithelial electrical resistance (TEER; a measure of barrier function) and integration of mechanical stretch or shear stress (256). The first study on SARS-CoV-2 infection in a bioengineered alveolus has employed an epithelial-endothelial co-culture approach under shear stress, which resulted in the differential expression of SARS-CoV-2 entry receptors as compared to 2D monolayers (99). Interestingly, infection with low SARS-CoV-2 titers induces a downregulation of NRP1 and ACE2 but an upregulation of TMPRSS2 expression in alveolar epithelial cells illustrating how multiple factors affect SARS-CoV-2 susceptibility and contribute to SARS-CoV-2 spread in the distal lung (99). In agreement with inefficient SARS-CoV-2 infection kinetics in alveolar cultures (13, 82), unproductive SARS-CoV-2 infection has also been observed in the alveolar layer in the LOC (99). However, SARS-CoV-2 infection elicits strikingly different responses among the cell types in co-culture. The alveolar cell layer remains largely intact, in line with previous findings in organoid models (101). In contrast, vascular injury is evident by 3 days post-infection resulting in damaged barrier function (99). This study provides evidence that the lung microvasculature is an essential contributor to model COVID-19 pathology *in vitro*, particularly in the maintenance of a prolonged pro-inflammatory response and IL-6 secretion (99). Disruption of the air-blood barrier in an alveolus-on-chip SARS-CoV-2 infection model and translocation of the virus to the vascular compartment has recently been demonstrated by another group, though the mechanism of vascular damage seems to be different (257). In the future, the application of LOC co-culture models will provide more mechanistic insights into host-pathogen interaction and inflammation-mediated damage. Moreover, LOC devices are superior to pure epithelial mono-cultures in predicting the protective effect of drugs on barrier integrity and inflammation [(99); Figure 2].

### Modeling Alveolar Epithelium Regeneration and COVID-19-Associated Fibrotic Tissue Remodeling

Most *in vitro* studies on COVID-19 have focused on the acute phase of the disease. However, uncontrolled inflammatory and early fibrotic signatures are typically found in post-mortem lung autopsies of COVID-19 patients after a long disease course (132, 258). Radiologic abnormalities, including small airway abnormalities, are also found in the lungs of recovering COVID-19 patients but their consequences on the patient's quality of life are of yet unknown (170). Therefore, it will be important to dissect the sequela of virus clearance, resolution of the immune response and tissue regeneration in more detail. Unfortunately, it may take years until large patient cohorts have been followed-up for a sufficient amount of time to conclude on the clinical course of fibrotic remodeling. Therefore, it is mandatory to experimentally study lung fibrosis in the aftermath of COVID-19 in dedicated SARS-CoV-2 infection models. For this purpose, a pre-requisite is a model system of the alveoli that supports multicellular composition including epithelial, mesenchymal and immune cells and remains stable over an extended period of time to monitor the acute phase of COVID-19 and subsequent progression to fibrosis.

Animal models provide the complete tissue microenvironment and systemic context for temporally controlled infection. Humanized ACE2 mice develop COVID-19 and pathologic tissue remodeling has been confirmed in their lungs ~3 days post-infection (217, 219, 220). These studies have been conducted in healthy young or old animals, however, other predispositions than age have not yet been investigated *in vivo* in the context of COVID-19. Multiple groups have demonstrated that viral infection exacerbates IPF in the bleomycin mouse model (259,261). It will be interesting to investigate COVID-19-associated fibrosis in bleomycin-induced or genetically predisposed (e.g., MUC5B, SP-C, and TERT) animal models. Treatment with anti-fibrotic therapy and prolonged monitoring will eventually identify effective candidate compounds and unravel the mechanisms of tissue regeneration after acute lung injury. Nevertheless, it is questionable that these findings can directly translate to human COVID-19 due to the fundamental differences in lung architecture, regeneration, and immune response [(262); Figure 2]. Pre-clinical drug testing is expected to be more accurate in macaques due to the phylogenic proximity to humans (228, 263). However, few animal experiments are performed in non-human primates due to logistic, financial, and ethical concerns posing the need for reliable *in vitro* infection models.

With intact alveolar structures and preserved local immune responses, PCLS from animals have proven their utility in the study of viral infection such as influenza A viruses (264). Although pulmonary explants of human proximal and distal airways are susceptible to SARS-CoV-2 infection (265), PCLS technique has not yet been used to investigate COVID-19. Hence, PCLS can be potentially employed to study the action of anti-fibrotic agents within SARS-CoV-2-infected alveolar epithelium. For instance, senolytic combination treatment (dasatinib and quercetin) possesses anti-fibrotic activity in mouse PCLS (266) and might be an interesting treatment strategy in the context of COVID-19 given the acquisition of a senescence-like phenotype in SARS-CoV-2 infected syncytial alveolar epithelial cells. Despite the applicability of PCLS for the screening of anti-fibrotic agents, the utility of this model is limited for this particular disease (267). PCLS only permits to study the local immune response, ignoring the important recruitment of circulating immune cells. In addition, short term of culture limits investigations over time (Figure 2).

Alternatively, pulmonary fibrosis has been studied in organoid models, which can be maintained in culture for several weeks and passages. hPSC-derived lung organoids that contain a mix of epithelial and mesenchymal cells have been genetically engineered to develop Hermansky-Pudlak syndrome, a clinical condition with similarities to IPF, and have been sequenced to discover potential new drug targets (205). Despite accurate recapitulation of the disease phenotype and a gene expression signature of early fibrosis, the fibrotic organoids lack some major hallmarks of IPF such as activation of TGF- signaling. Transcriptomic and histologic alterations of IPF have recently been modeled in hPSC-derived ATII-like cell cultures maintained in ALI (207). Given that alveolar epithelial progenitors, an essential contributor of aberrant wound healing, are enriched in hPSC-derived models they have been suggested as suitable model to study pulmonary fibrosis (207). However, since fibrosis predominantly occurs in senescent lungs, it is suboptimal to study fibrosis in an embryonic stem cell derived model system. Therefore, it will be interesting to study fibrotic changes in mixed mature long-term organoid culture after SARS-CoV-2 infection.

Potentially more physiologically relevant are ALI models, that can mimic SARS-CoV-2 infection by inhalation and provide a scaffold for epithelial/mesenchymal co-cultures. Yet it is not easy to avoid transdifferentiation of ATII cells, the main alveolar target cells of SARS-CoV-2 and drivers of fibrosis. Recently, the EpiAlveolar 3D tissue culture system has been developed to model micro-particle inhalation and the resulting pro-fibrotic events in the alveolar epithelium [(268); Figure 2]. Repeated exposure over an extended time period is possible, which implies that these models could also be applied to study the progression of acute COVID-19 to pulmonary fibrosis. However, it remains to be tested how efficiently SARS-CoV-2 infects these 3D alveolar tissue culture models and whether immune cells are required to induce fibrosis.

A higher degree of complexity can be achieved in LOC models. In the future, they might enable the investigation of cellular, mechanical, and chemical processes resulting in complex multi-factorial diseases such as pulmonary fibrosis. As compared to conventional cell culture, LOC models support stable cell culture systems over an extended time period, which is particularly relevant for studying pulmonary fibrosis, a disease of older age. However, as compared to the *in vivo* situation, long-term culture of cells in microfluidic devices remains a limiting factor to date. Nevertheless, aspects of lung inflammatory and fibrotic processes can be studied under nearly physiological conditions in such microfluidic devices. Importantly, it has been shown that substrate stiffness, porosity, and physiologic stretch has to be taken into account when studying tissue regeneration and assessing efficacy of therapeutic compounds (50). In addition, LOC models are well-suited to establish co-cultures enabling to study the sequence of molecular events in COVID-19 at the delicate air-blood barrier in the presence of alveolar resident cell types and peripheral immune cells (Figure 2). Taken together, advanced *in vitro* models have the potential to provide highly relevant data to discover novel effective treatment strategies for COVID-19, identify predictive biomarkers for a severe course of the disease and elucidate the mechanisms of lung repair in recovering patients.

## Conclusion

Refined treatment strategies for recently emerged SARS-CoV-2 are becoming available and have improved the management of COVID-19 patients. Evidence has been gathered that particularly critically ill COVID-19 patients suffer from pulmonary dysfunction even months after diagnosis and may possibly never fully recover. SARS-CoV-2 infection of alveolar epithelial cells and an imbalanced inflammatory response result in DAD and trigger a fibrotic response to regenerate the epithelial barrier and lung function. To date, it is not clear whether fibrosis will develop and consequently resolve or progress. IPF, the most severe form of interstitial pulmonary fibrosis, is a fatal disease and few treatment options exist to slow down the progression of chronic fibrosis. It is therefore crucial, to delineate the early mechanisms that drive fibrotic progression after virus clearance. *In vivo* models capture aspects of human COVID-19 and have been classically used to study progression of IPF. However, due to inter-species anatomical and immunological differences, the translation of basic and pre-clinical animal research to patient-relevant insights often fails (182). The systemic manifestation of COVID-19 and the complex host-pathogen interactions highlight the importance of human-derived models to study the underlying mechanisms of the disease. Lung organoids promote cell-cell and cell-matrix interactions and provide a robust SARS-CoV-2 infection model of the alveoli. Due to their capacity to propagate and the relatively cost-efficient culture methods, patient-derived organoids enable medium-throughput drug screening for precision medicine in cancer and other diseases (269, 270). We expect that personalized therapy will also improve the management of severely ill COVID-19 patients. However, organoids are not ideally suited to construct complex co-cultures or study biomechanical forces, which might be a pre-requisite to gain more insights into COVID-19-associated pulmonary fibrosis. In this regard, PCLS, 3D *in vitro* tissue culture, and LOC are complementary models to lung organoids providing a higher degree of microenvironmental cues. These models have been used successfully to study IPF and are expectedly permissive for SARS-CoV-2 infection. Moreover, LOC holds the unique opportunity to study SARS-CoV-2 mediated alveolar injury at ALI and under stretch. It is well-known that stretch can significantly alter epithelial barrier permeability (215), ATII cell function and tissue regeneration (41, 271, 272). However, studies about the impact of stretch on the acute and recovery phase of COVID-19 are still lacking. The combinational application of advanced *in vitro* models is expected to generate meaningful data on the molecular processes taking place in the lungs of COVID-19 patients and providing insights into disease course before patient data from large cohorts become available. Hopefully, this knowledge will help to improve patient care and prevent fibrosis at an early stage of progression.

## Author Contributions

MK, NR, CM, AS, TG, OG, MF-C, NH, and MK-dJ contributed to the review, writing, and revising the manuscript. All authors contributed to the article and approved the submitted version.

## Conflict of Interest

NR and NH are employed by AlveoliX AG. OG and TG are shareholder and in the scientific board of AlveoliX AG. MK and MK-dJ are collaborators of AlveoliX AG. The remaining authors declare that the research was conducted in the absence of any commercial or financial relationships that could be construed as a potential conflict of interest.

## Abbreviations

-SMA: -smooth muscle actin
ACE2: angiotensin-converting enzyme 2
ALI: air-liquid interface
AM: alveolar macrophage
APN: aminopeptidase N
ARDS: Acute Respiratory Distress Syndrome
ATI: type I alveolar epithelial cell
ATII: type II alveolar epithelial cell
COVID-19: coronavirus disease 2019
CT scan: computed tomography scan
DAD: diffuse alveolar damage
DPP4: dipeptidyl peptidase 4
ECM: extracellular matrix
FITC: fluorescein isothiocyanate
hPSC: human pluripotent stem cell
IFN: interferon
IL: interleukin
ILD: interstitial lung disease
IPF: idiopathic pulmonary fibrosis
LOC: lung-on-chip
MERS: Middle East Respiratory Syndrome
MERS-CoV: Middle East Respiratory Syndrome coronavirus
MUC5B: mucin-5B
NRP1: neuropilin-1
NRP2: neuropilin-2
PCLS: precision-cut lung slices
PCSK: proprotein convertase subtilisin kexin
PDGF: platelet-derived growth factor
RBD: receptor-binding domain
RTC: replication and transcription complex
S protein: spike protein
SARS: Severe Acute Respiratory Syndrome
SARS-CoV: Severe Acute Respiratory Syndrome coronavirus
SARS-CoV-2: Severe Acute Respiratory Syndrome coronavirus 2
SP-A: surfactant protein A
SP-C: surfactant protein C
TEER: transepithelial electrical resistance
TERC: telomerase RNA component
TERF-1: telomeric repeat-binding factor 1
TERT: telomerase reverse transcriptase
TGF-: transforming growth factor
TLC: total lung capacity
TMPRSS2: transmembrane protease serine subtype 2
TNF-: tumor necrosis factor .
